# Peroxiredoxin 1 inhibits streptozotocin-induced Alzheimer’s disease-like pathology in hippocampal neuronal cells via the blocking of Ca^2+^/Calpain/Cdk5-mediated mitochondrial fragmentation

**DOI:** 10.1038/s41598-024-66256-x

**Published:** 2024-07-08

**Authors:** Junghyung Park, Jinyoung Won, Eunyeoung Yang, Jincheol Seo, Jiyeon Cho, Jung Bae Seong, Hyeon-Gu Yeo, Keonwoo Kim, Yu Gyeong Kim, Minji Kim, Chang-Yeop Jeon, Kyung Seob Lim, Dong-Seok Lee, Youngjeon Lee

**Affiliations:** 1 National Primate Research Center, Korea Research Institute of Bioscience and Biotechnology (KRIBB), Cheongju, Republic of Korea; 2 Department of Life Science, University of Seoul, Seoul, Republic of Korea; 3 KRIBB School of Bioscience, Korea University of Science and Technology (UST), Daejeon, Republic of Korea; 4 School of Life Sciences, BK21 FOUR KNU Creative BioResearch Group, Kyungpook National University, Daegu, Republic of Korea; 5 Department of Bio and Brain Engineering, Korea Advanced Institute of Science and Technology (KAIST), Daejeon, Republic of Korea; 6 Futuristic Animal Resource & Research Center, Korea Research Institute of Bioscience and Biotechnology (KRIBB), Cheongju, Republic of Korea

**Keywords:** Peroxiredoxin 1(Prx1), Oxidative stress, Alzheimer’s disease (AD), Streptozotocin, Calpain, Mitochondria, Neurochemistry, Cell signalling, Organelles, Post-translational modifications, Cellular neuroscience, Molecular neuroscience

## Abstract

Oxidative stress plays an essential role in the progression of Alzheimer’s disease (AD), the most common age-related neurodegenerative disorder. Streptozotocin (STZ)-induced abnormal brain insulin signaling and oxidative stress play crucial roles in the progression of Alzheimer’s disease (AD)-like pathology. Peroxiredoxins (Prxs) are associated with protection from neuronal death induced by oxidative stress. However, the molecular mechanisms underlying Prxs on STZ-induced progression of AD in the hippocampal neurons are not yet fully understood. Here, we evaluated whether Peroxiredoxin 1 (Prx1) affects STZ-induced AD-like pathology and cellular toxicity. Prx1 expression was increased by STZ treatment in the hippocampus cell line, HT-22 cells. We evaluated whether Prx1 affects STZ-induced HT-22 cells using overexpression. Prx1 successfully protected the forms of STZ-induced AD-like pathology, such as neuronal apoptosis, synaptic loss, and tau phosphorylation. Moreover, Prx1 suppressed the STZ-induced increase of mitochondrial dysfunction and fragmentation by down-regulating Drp1 phosphorylation and mitochondrial location. Prx1 plays a role in an upstream signal pathway of Drp1 phosphorylation, cyclin-dependent kinase 5 (Cdk5) by inhibiting the STZ-induced conversion of p35 to p25. We found that STZ-induced of intracellular Ca^2+^ accumulation was an important modulator of AD-like pathology progression by regulating Ca^2+^-mediated Calpain activation, and Prx1 down-regulated STZ-induced intracellular Ca^2+^ accumulation and Ca^2+^-mediated Calpain activation. Finally, we identified that Prx1 antioxidant capacity affected Ca^2+^/Calpain/Cdk5-mediated AD-like pathology progress. Therefore, these findings demonstrated that Prx1 is a key factor in STZ-induced hippocampal neuronal death through inhibition of Ca^2+^/Calpain/Cdk5-mediated mitochondrial dysfunction by protecting against oxidative stress.

## Introduction

Alzheimer’s disease (AD) is a progressive neurodegenerative disease characterized by continuing memory impairments cognitive declines, and changes in behavior and personality. Abnormal accumulation of amyloid plaques and hyperphosphorylated tau in the form of neurofibrillary tangles, and oxidative stress are among the most prominent pathological features in AD brains^1^. Insulin signaling in the brain contributes to the maintenance of numerous brain functions, such as nutrient homeostasis, reproduction, cognition, and memory. Therefore, alteration of insulin signaling in the brain is closely associated with brain aging, and neurodegeneration^2^. Impaired brain insulin signaling is observed in the brains of AD patients and experimental models^3^. Additionally, anti-diabetic drugs exert protective effects on AD-related pathologies in the AD models of mice and monkeys^4,5^. Streptozotocin (STZ) is a diabetogenic and toxic compound for pancreatic β-cell generally used to establish animal models of diabetes owing to its ability to selectively disrupt the insulin signaling pathway^6^. The intracerebroventricular (ICV) injection of STZ into the brain is a well-established non-transgenic AD animal model showing behavioral, pathological, and molecular aspects of AD, including memory impairment, neuronal loss, and oxidative stress^7,8^.

Oxidative stress is caused by an imbalance between the production of reactive oxygen species (ROS) and antioxidant defense and plays a critical role in the progression of AD^9,10^. The central nervous system (CNS) is particularly susceptible to oxidative stress because of its high demand for and consumption of oxygen, and high concentrations of easily oxidized lipid-rich material^11^. Peroxiredoxins (Prxs) are a family of antioxidant enzymes that eliminate hydrogen peroxide (H_2_O_2_) and is related to various cellular signal transduction processes by maintaining the redox state balance in various types of neuronal cells^12,13^. There are six mammalian Prxs, classified into three subtypes (typical 2-Cys, atypical 2-Cys, and typical 1-Cys) depending on their catalytic mechanism of peroxide reduction. Prx1 belongs to the 2-Cys Prx and is widely distributed within diverse subcellular compartments, including the cytosol and nucleus^14^. Prx1 expression level was elevated in the brains of patients with AD^15,16^, whereas it was decreased in other research^17^. Prx1 exerts protective effects in experimental models for AD^18^. However, molecular mechanisms of Prx1 on the protection of AD progression are still lacking.

Mitochondria are an essential cellular organelle with key regulatory functions in energy production, oxidative balance, and calcium homeostasis^19^. Several molecular abnormalities involving mitochondria, such as loss of energy metabolism, abnormal morphology, and accumulation of ROS were observed in the brain of patients and animal models with AD^20–24^. Mitochondria are highly dynamic subcellular organelles that constantly repeat the process of fusion and fission events. The balance of mitochondrial fission and fusion is crucial for cellular processes in hippocampal neurons^25,26^. Cumulative mitochondrial fission induces mitochondrial fragmentation and is closely associated with pathogenesis of STZ-induced AD models^27,28^. Dynamin-1-like protein (Drp1) is a key regulator of mitochondrial fission. It is recruited from the cytosol to mitochondria, then triggers mitochondrial division by increasing their activity^29,30^. The activity of Drp1 is controlled by post-translational modification, including phosphorylation^31^. In particular, the phosphorylation of Drp1 at Ser616 (S616) has been shown to control the activity of Drp1, which plays an important role in various neurodegenerative pathological processes^32,33^. However, the molecular relationship between oxidative stress and changes in mitochondrial dynamics induced by STZ in an AD experimental model has not been fully elucidated.

Calpains are calcium (Ca^2+^)-dependent cysteine proteinases that are involved in multiple cellular functions, such as proliferation, differentiation, growth, and apoptosis^34^. Abnormal activation of calpains induced by disruption of Ca^2+^ homeostasis is observed around amyloid plaque and neurofibrillary tangles of AD patients and experimental models^35,36^, and inhibition of calpains improved spatial-working memory and synaptic transmission in AD mice model^37,38^. In particular, the elevated expression level of calpain-2 (m-calpain), one of the major calpain isoforms, is known to reflect calpain activity in neuronal cells and is closely associated with the progression of AD pathologies^39–41^.

Cyclin-dependent kinase 5 (Cdk5) belongs to the family of proline-directed serine/threonine kinase and is activated by the neuron-specific activator p35 under normal conditions, and can be deregulated by p25, which is the cleaved form of p35^42^. Cdk5 performs an important role in the maintenance of neuronal development, survival, synaptic plasticity, and neurotransmission^43^. However, uncontrolled activity of Cdk5/p25 is involved in the development of AD pathologies, including tau phosphorylation^44^, and amyloidogenesis^45^. Therefore, preservation of Cdk5 homeostasis is suggested as a reasonable therapeutic target for ameliorating AD pathological processes^46,47^. Interestingly, Cdk5 was also identified as an upstream regulator of mitochondrial fission via phosphorylation of Drp1 at S616 in neurodegenerative conditions^48,49^. Calpain is one of the factors involved in the cleavage of p25, which has been linked to AD pathogenesis^37^.

In this study, we evaluated whether oxidative stress-induced Ca^2+^ accumulation affects the calpain-2-mediated progression of AD-like pathologies in STZ-induced hippocampal neuronal HT-22 cells. Therefore, we focused on Prx1 as a regulator of STZ-induced oxidative stress. Furthermore, to validate the effect of Prx1 on the molecular signal pathway in the progression of STZ-induced AD-like pathologies (neuronal apoptosis, synaptic function, tau pathology, and mitochondrial fragmentation), we determined the change in calpain, cdk5, and mitochondria morphology using antioxidant molecule and Ca^2+^ chelator. These studies suggested that maintenance of oxidative balance via an increase in antioxidant defense is a promising target to ameliorate the progress of AD.

## Materials and methods

### Cell culture and treatment

HT-22 cells were derived from HT-4 cells, which were immortalized from primary mouse hippocampal neuronal culture^50^. HT-22 cells were maintained at 37 °C in DMEM with high glucose (Welgene, Daegu, Korea) supplemented with 10% FBS (Thermo Fisher Scientific, Waltham, MA, USA), 100 U/ml penicillin, and 100 μg/ml streptomycin (Welgene) in a humidified atmosphere incubator (Thermo Fisher Scientific) with 5% CO_2_. The cells were pretreated with BAPTA-AM (0.5 μM; Thermo Fisher Scientific), NAC (5 mM; Sigma-Aldrich, St. Louis, MO, USA) for 30 min and were incubated with STZ (10 mM; Sigma-Aldrich).

## Plasmid construction preparation of stable cell line

Prx1 gene including plasmid (pLenti6.3-Prx1-V5; Thermo Fisher Scientific) kindly provided by Dr. Dong-Seok Lee (Kyungpook National University, Daegu, Korea). Prx1 was amplified by PCR with LA Taq polymerase (TaKaRa, Shiga, Japan). These genes were cloned into a gateway entry vector pCR8/GW/TOPO (Thermo Fisher Scientific) to generate expression clones by performing LR recombination between the entry vector and gateway destination vectors pLenti6.3/V5-DEST (Thermo Fisher Scientific). pLenti6.3/V5-DEST vector has a C-terminal V5 epitope that aids in detecting recombinant proteins during immunoblotting analysis^51^. The sequences of the constructed vectors were confirmed by performing restriction mapping and DNA sequencing. 1 μg of pLenti6.3-Prx1-V5 plasmid was transfected into the HT-22 cells by using effectene (Qiagen, Hilden, Germany), according to the manufacturer's instructions. After 24 h, the transfected cells were selected using 8 μg/mL blasticidin (Thermo Scientific).

## Western blot analysis

Whole protein lysates were prepared using the PRO-PREP protein extraction solution (Intron Biotechnology, Seongnam, Korea), and mitochondrial fractions were performed with a mitochondria isolation kit (Thermo Fisher Scientific). Equal amounts of proteins were separated by electrophoresis on 8–15% SDS-PAGE gels and transferred onto nitrocellulose membranes (BD Biosciences, NJ, USA). The membranes were cut according to the expected molecular weight of the target protein before antibody hybridization. The membranes were blocked by incubation in blocking buffer (BD Biosciences) and probed with the following antibodies overnight at 4 °C: anti-Prx1 (Ab Frontier, Seoul, Korea), anti-β-actin (Sigma-Aldrich), anti-V5, anti-AT8 (Thermo Fisher Scientific), anti-cleaved caspase-3, anti-PARP, p-Tau(S262), anti-Drp1, anti-p-Drp1(S616), anti-COXIV, anti-Cdk5 (Cell Signaling, MA, USA), anti-NeuN, anti-PSD95, (Abcam, MA, USA), anti-p35, anti-calpain-2 (Santa Cruz Biotechnology, Dallas, TX, USA). The membranes were washed with TBS with 0.1% Tween-20 (TBST) and incubated with horseradish peroxidase-conjugated secondary antibodies (Cell Signaling) for 1 h at room temperature. After washing with TBST, protein bands were visualized using enhanced chemiluminescence reagent in the Chemi DocXRS + imaging system (Bio-Rad, Hercules, CA, USA). Finally, densitometric analysis was performed using Image Lab software, version 3.0 (Bio-Rad).

## MTT assay

Mock- and Prx1-expressed HT-22 cells were cultured on 96-well plates for 24 h. After experiments, each cell sample was incubated for 30 min at 37 °C with MTT (0.5 mg/mL; Sigma-Aldrich), and then 100 μL DMSO (Sigma-Aldrich) was added. Absorbance was measured at 550 nm.

## Measurements of intracellular ATP levels

The intracellular ATP levels were determined using an ATP determination kit (Thermo Fisher Scientific) following the manufacturer’s instructions. After experiments, whole protein lysates at a concentration of 1 μg/mL were used for the measurements of intracellular ATP levels.

## Mitochondrial imaging

To observe mitochondrial morphology, DsRed2-mito including plasmid (pLenti6.3-DsRed2-mito) was kindly provided by Dr. Dong-Seok Lee (Kyungpook National University, Daegu, Korea). DsRed2-mito expressed HT-22 cells were seeded on 0.01% poly-D-lysine-coated round coverslips and were incubated for 24 h. After experiments, cells were washed with PBS, and fixed with 4% paraformaldehyde for 1 h. After washing, the coverslips were mounted on slides with mounting medium (VECTOR Laboratories, CA, USA). To observe mitochondrial morphology in Prx1-expressed HT-22 cells, MitoTracker Green (100 µM; Thermo Fisher Scientific) was incubated with the cells. Mitochondrial images were acquired using the LSM-710 confocal microscope (Carl Zeiss, Jena, Germany). Mitochondrial length measurements were performed using Image J software as previously described^52^. Briefly, average mitochondrial length was evaluated from more than 30 mitochondria particles per cell in over 10 cells.

## Determination of intracellular Ca^2+^ levels

Intracellular Ca^2+^ level was measured using Fluo-4 AM (Thermo Fisher Scientific). After experiment, cells were incubated with Fluo-4 AM for 30 min at 37 °C. After washing with HBSS, images of Fluo-4 AM were obtained using an ECLIPSE Ti-U microscope (Nikon, Tokyo, Japan), and the fluorescence intensity of Fluo-4 AM was measured with image J software.

## Measurements of intracellular ROS

Intracellular ROS generation was assessed using CM-H_2_DCFDA. After experiments, cells were incubated with 5 μM CM-H_2_DCFDA (Thermo Fisher Scientific) for 30 min at 37 °C, and then analyzed using an ECLIPSE Ti-U microscope (Nikon) and FACSCalibur flow cytometry (BD Biosciences).

## Statistical analysis

The data represent the mean ± SD from three independent experiments (n ≥ 3). Experimental differences were tested for statistical significance using GraphPad Prism 9 software (San Diego, CA, USA). Multiple group analyses were performed by one-way analysis of variance (ANOVA) followed by Tukey’s post hoc test for normally distributed datasets. Statistical significance was set at *p* < 0.05, and is indicated on the graphs using asterisks; *P*-values < 0.01 and < 0.001 were indicated by two and three asterisks, respectively.

## Results

### STZ-mediated Prx1 induction inhibits the progression of AD-like pathology

**Figure 1:**
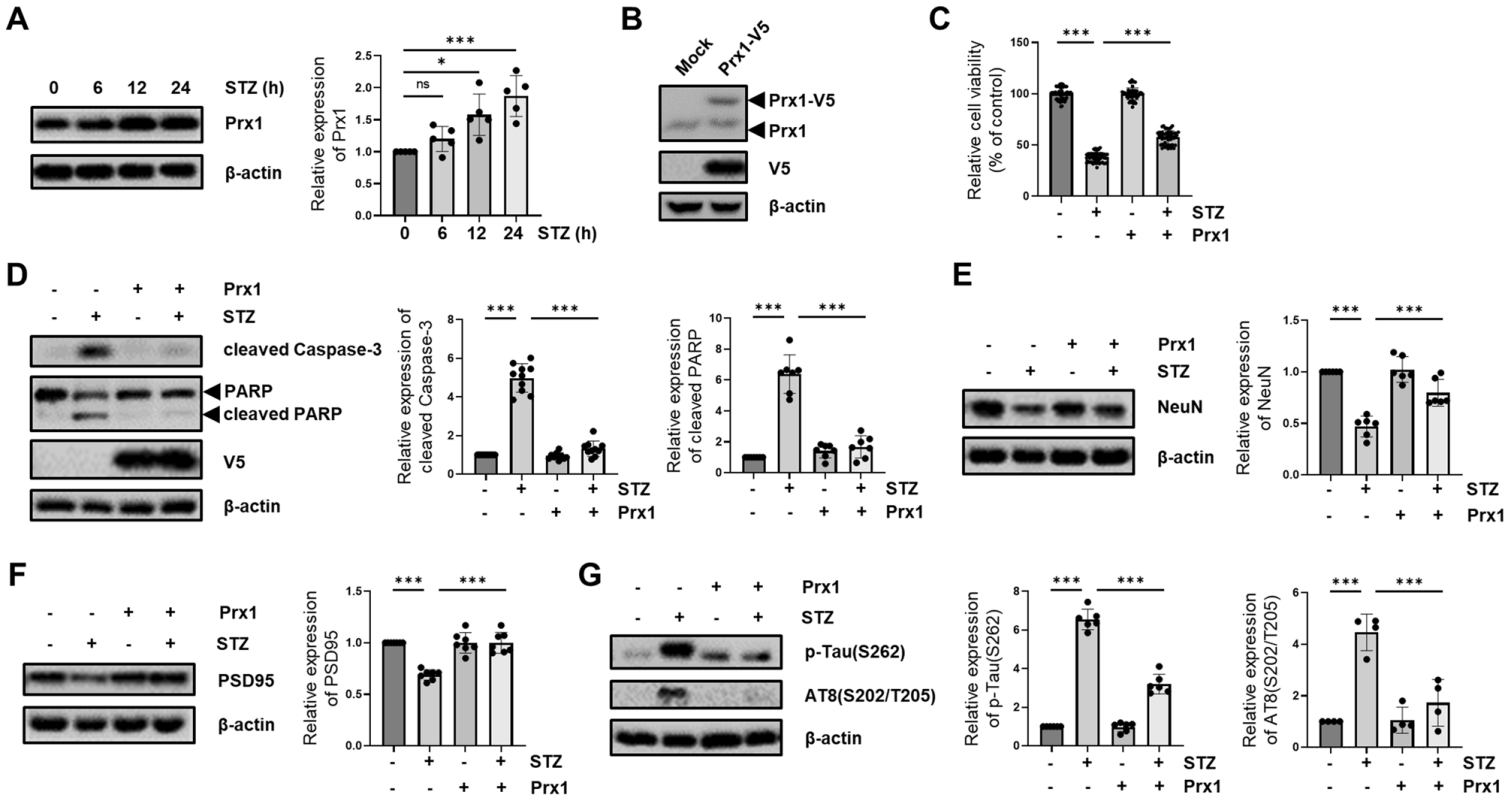
Effect of Prx1 on STZ-induced AD-like pathology. (**A**) The protein expression level of Prx1 was determined by western blotting in STZ (10 mM)-treated HT-22 cells following indicated time points (6–24 h). (**B**) Prx1-expressed HT-22 cells were verified using western blotting with Prx1 and V5-tag antibodies. (**C**) Cell viability was determined using the MTT assay in mock- and Prx1-expressed HT-22 cells according to STZ treatment for 24 h. (**D**) Cleaved Caspase-3, cleaved PARP, (**E**) NeuN, (**F**) PSD95, and (**G**) p-Tau (S262), AT8 (S202/T205) protein expression level were confirmed by western blotting in STZ (24 h)-treated mock- and Prx1-expressed HT-22 cells. Blots were cropped to highlight the region of interest; full blot images in this article are provided in supplementary information. The data are presented as mean values ± SD (n ≥ 3). * denotes *p* < 0.05, ** denotes *p* < 0.01, and *** denotes *p* < 0.001.

We investigated the Prx1 protein expression levels in HT-22 cells following time-dependent STZ treatments. STZ concentration (10 mM) referred to induce AD-like pathology in HT-22 cells^53^. We found that Prx1 protein expression level was gradually increased after 12 h STZ treatments (Fig. 1A). To assess the effect of inducible Prx1 expression by STZ on the process of AD-like pathology, we generated V5-tagged Prx1 (Prx1-V5) stably expressing HT-22 cells line by using transfection of pLtneti6.3-Prx1-V5. We identified the exogenous expression of Prx1 (Prx1-V5) by western blotting using Prx1 and V5-tag antibodies (Fig. 1B). Prx1 expression reversed STZ-induced decreased cell viability and increased apoptotic markers, such as cleaved caspase-3 and cleaved PARP (Fig. 1C,D). Furthermore, we determined the effect of Prx1 on STZ-induced neuronal loss and synaptic function using immunoblotting with NeuN (neuronal marker), and PSD95 (post-synapse marker). Our results showed that STZ-induced down-regulation of NeuN and PSD95 were inhibited in Prx1 expression (Fig. 1E,F). We also confirmed that Prx1 impacted the phosphorylation of tau epitopes, such as p-Tau(S262) and AT8(S202/T205). Up-regulated p-Tau (S262) and AT8(S202/T205) by STZ treatment were also suppressed by Prx1 expression (Fig. 1G). These results suggested that Prx1 induction by STZ regulates the STZ-induced process of AD-like pathology, apoptotic neuronal loss, synaptic loss, and tauopathy in hippocampal cell lines.

### Prx1 prevents STZ-induced mitochondrial fragmentation and dysfunction

**Figure 2:**
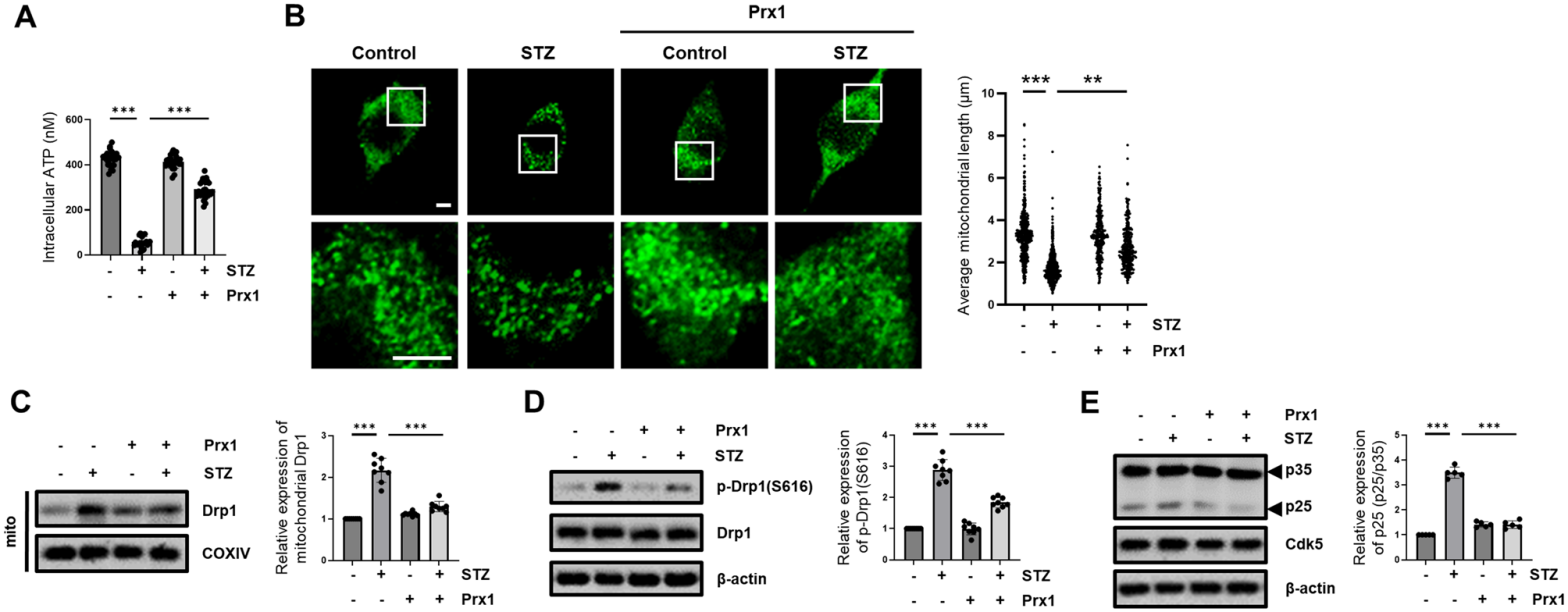
Effect of Prx1 on STZ-induced mitochondrial dysfunction and change in mitochondrial morphology. (**A**) Intracellular ATP levels were measured using the ATP determination kit in STZ (12 h)-treated mock- and Prx1-expressed HT-22 cells. (**B**) Mitochondrial morphology was observed with mitotracker (100 μM) staining using confocal microscopy in STZ (12 h)-treated HT-22 and Prx1-expressed cells. The bottom panels showed the magnified images of regions indicated by white squares in the top panels; scale bar, 5 μm. The graph showed distribution of all mitochondrial particles and average mitochondrial length. (**C**) Mitochondrial location of Drp1 was analyzed by western blotting from mitochondrial isolated protein in mock- and Prx1-expressed HT-22 cells treated with STZ for 12 h. COXIV was used as the loading control for the mitochondria. (**D**) The protein expression levels of p-Drp1(S616) and Drp1 were determined using western blot analysis in STZ (12 h)-treated mock- and Prx1-expressed HT-22 cells. Drp1 was used as the loading control for p-Drp1(S616). (**E**) Protein levels of p25/35 and Cdk5 in STZ (6 h)-treated mock- and Prx1-expressed HT-22 cells were confirmed using western blotting. Blots were cropped to highlight the region of interest; full blot images in this article are provided in supplementary information. The data are presented as mean values ± SD (n ≥ 3). ** denotes *p* < 0.01, and *** denotes *p* < 0.001.

We previously reported that changes in mitochondrial morphology and mitochondrial function relate to STZ-induced progression of AD-like pathology^53^. Therefore, we measured changes in intracellular ATP levels after STZ treatment for 12 h in mock- and Prx1-expressed HT-22 cells. Our results indicated that the down-regulated intracellular ATP level by STZ treatment was suppressed by Prx1 expression (Fig. 2A). We determined whether Prx1 affects STZ-induced mitochondrial fragmentation. To observe changes in mitochondrial morphology after STZ treatment for 12 h in HT-22 and Prx1-expressed HT-22 cells, we stained with mitotracker green after experiments. Our result indicated that the decrease of mitochondrial average length by STZ treatment in HT-22 cells was inhibited in Prx1-expressed HT-22 cells (Fig. 2B,C). STZ-induced mitochondrial fragmentation depended on the Drp1 translocation from the cytoplasm to the mitochondria by phosphorylation of Drp1(S616) via activating Cdk5/p25 signaling pathway^53^. Therefore, we assess the effects of Prx1 on the Cdk5/p25-mediated Drp1 activation by STZ treatment for 12 h. Our results indicated that the STZ-induced increase in the level of mitochondrial Drp1 was inhibited by Prx1 expression (Fig. 2C). Drp1(S616) phosphorylation was increased by STZ treatment for 12 h, and up-regulated Drp1(S616) phosphorylation was repressed by Prx1 expression (Fig. 2D). Moreover, increased level of p25 by STZ treatment for 6 h, which is known to show the highest level of p25 by STZ treatment in HT-22 cells^53^, was inhibited by Prx1 expression (Fig. 2E). Therefore, we demonstrate that induction of Prx1 by STZ regulates Cdk5-mediated mitochondrial fragmentation and dysfunction by inhibiting cleavage of p35 to p25.

### Prx1 regulates STZ-induce Ca^2+^-mediated mitochondrial fragmentation and dysfunction through calpain/Cdk5-mediated Drp1 activation

**Figure 3:**
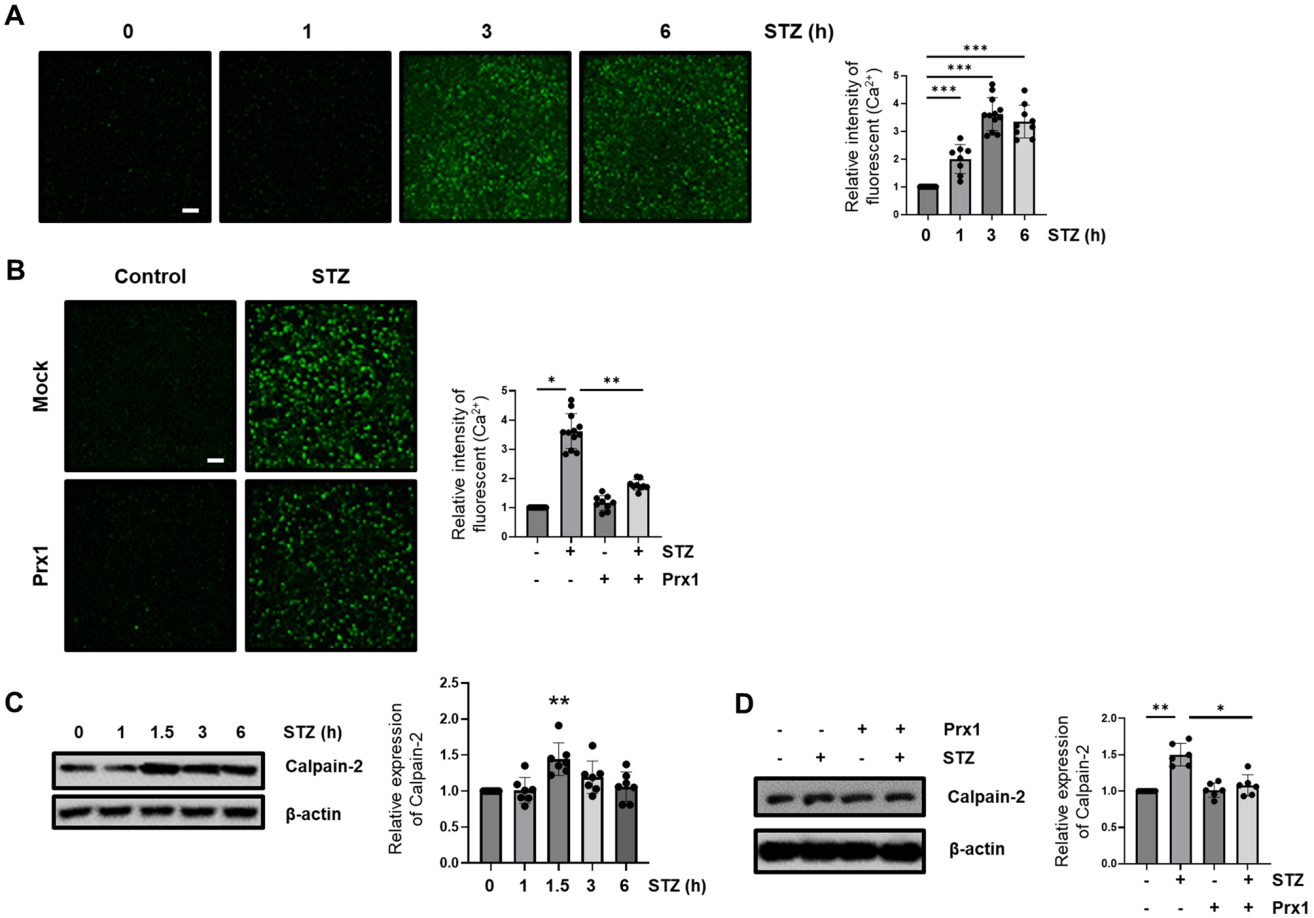
Effect of Prx1 on the STZ-induced Ca^2+^/Calpain signaling. (**A**) Intracellular Ca^2+^ level in STZ-treated HT-22 cells following indicated time points (1–6 h) was observed with Fluo-4 AM (5 μM) staining using fluorescent microscopy; scale bar, 200 μm. Graph showing Fluo-4 AM fluorescent spectrophotometer results. (**B**) Intracellular Ca^2+^ levels in STZ (3 h)-treated HT-22 and Prx1-expressed HT-22 cells with Fluo-4 AM observed using fluorescent microscopy; scale bar, 200 μm. Fluorescent spectrophotometer results of Fluo-4 AM represented as graphs. (**C**) Calpain-2 protein expression was confirmed by western blotting in HT-22 cells following indicated time point (1–6 h) treated STZ. (**D**) Calpain-2 expression was confirmed using western blotting in STZ (1.5h)-treated HT-22 and Prx1-expressed HT-22 cells. Blots were cropped to highlight the region of interest; full blot images in this article are provided in supplementary information. The data are presented as mean values ± SD (n ≥ 3). * denotes *p* < 0.05, ** denotes *p* < 0.01, and *** denotes *p* < 0.001.

**Figure 4:**
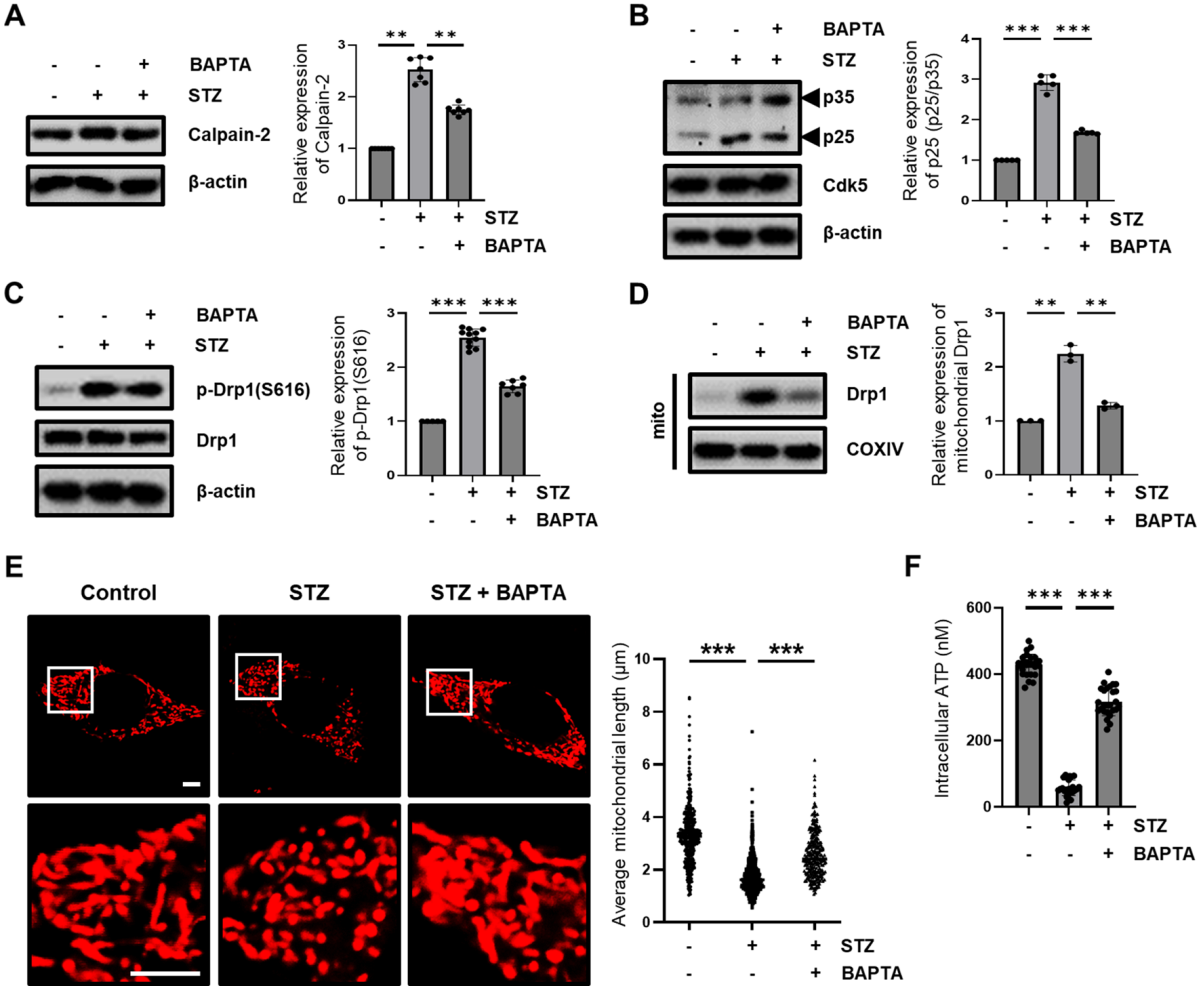
Effect of Ca^2+^ on STZ-induced calpain/Cdk5/Drp1 activation. (**A**) Calpain-2 protein expression level in STZ (1.5 h)-treated HT-22 cells pretreated with or without BAPTA-AM (0.5 μM) were confirmed with western blotting. (**B**) The proteins expression level of p25/35 and Cdk5 in STZ (6 h)-treated HT-22 cells pretreated with or without BAPTA-AM were confirmed with western blotting. (**C**) p-Drp1(S616), and (**D**) Mitochondrial Drp1 proteins expression level in STZ (12 h)-treated HT-22 cells pretreated with or without BAPTA-AM were confirmed with western blotting. COXIV was used as the loading control for the mitochondria. (**E**) Change in mitochondrial morphology was observed using confocal microscopy in STZ (12 h)-treated DsRed2-mito expressed HT-22 cells pretreated with or without BAPTA-AM. The bottom panels showed the magnified images of regions indicated by white squares in the top panels; scale bar, 5 μm. The graph showed distribution of all mitochondrial particles and average mitochondrial length. (**F**) Intracellular ATP levels were measured in STZ (24 h)-treated HT-22 cells pretreated with or without BAPTA-AM. The data are presented as mean values ± SD (n ≥ 3). ** denotes *p* < 0.01, and *** denotes *p* < 0.001.

Previous reports indicated that Prx5 inhibits the accumulation of intracellular Ca^2+^, calpain activation, and Cdk5 activation in an amyloid-beta (Aβ) oligomer-mediated AD cellular model^54^. Therefore, we measured intracellular Ca^2+^ levels using the intracellular Ca^2+^ indicator Fluo-4 AM, in time-dependent STZ-treated HT-22 cells. Intracellular Ca^2+^ was measured from 3 h STZ treatment and maintained to 6 h (Fig. 3A). An increased level of STZ-induced intracellular Ca^2+^ was down-regulated by Prx1 expression (Fig. 3B). The elevated expression level of calpain-2 is known to reflect calpain activation in neuron cells^40,41^. Thus, we then assessed whether Prx1 affected calpain-2 expression. We found that calpain-2 expression level was the highest at 1.5 h STZ treatment time, and was gradually down-regulated to 6 h (Fig. 3C). Prx1 inhibited STZ-induced increase of calpain-2 expression (Fig. 3D). We then assessed whether Prx1 influenced STZ-induced mitochondrial fragmentation through intracellular Ca^2+^ regulation using an intracellular Ca^2+^ chelator, BAPTA-AM. We found that inhibition of STZ-induced Ca^2+^ accumulation prevented the calpain activation and Cdk5 activation by p35 cleavage to p25 (Fig. 4A,B). Furthermore, STZ-induced increases of fragmented mitochondria and mitochondrial dysfunction were restored by intracellular Ca^2+^ chelation via controlling Drp1 activation (Fig. 4C,D,E,F). These results suggest that Prx1 regulates calpain/Cdk5-mediated mitochondrial fragmentation by controlling STZ-induced intracellular Ca^2+^ accumulation.

### Effect of intracellular Ca^2+^ on the STZ-induced AD-like pathology

**Figure 5:**
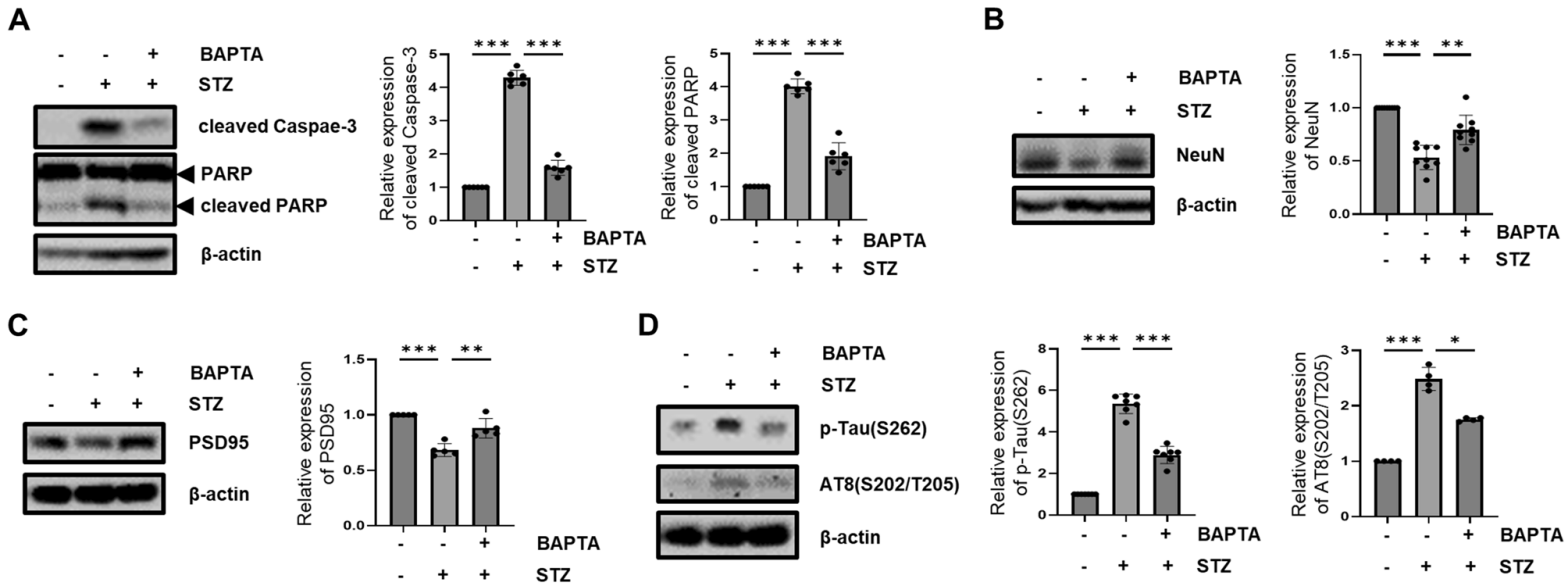
Effect of Ca^2+^ on STZ-induced AD-like pathology. (**A**) Cleaved Caspase-3 and cleaved PARP, (**B**) NeuN, (**C**) PSD95, (D) p-Tau(S262) and AT8(S202/T205) protein expression level were confirmed by western blotting analysis in STZ (24 h)-treated HT-22 cells pretreated with or without BAPTA. Blots were cropped to highlight the region of interest; full blot images in this article are provided in supplementary information. The data are presented as mean values ± SD (n ≥ 3). * denotes *p* < 0.05, ** denotes *p* < 0.01, and *** denotes *p* < 0.001.

Past demonstrations indicated that intracellular Ca^2+^ or control of mitochondrial morphology were key mediators in AD^54^. As we proved the role of Ca^2+^ as a regulator of STZ-induced mitochondrial fragmentation, we determined the effect of STZ-induced Ca^2+^ on the progression of AD-like pathology. STZ-induced Ca^2+^ inhibition with BAPTA-AM suppressed increased neuronal apoptosis and neuronal loss. The increased level of cleaved caspase-3 and cleaved PARP, reduced the level of NeuN by STZ reversed by BAPTA-AM treatment (Fig. 5A,B). In addition, the STZ-induced increased synaptic loss and Tau activation were confirmed with PSD95, p-Tau(S262), and AT8(S202/T205). STZ-induced decrease of PSD95 and increase of p-Tau(S262) and AT8(S202/T205) were attenuated by BAPTA-AM treatment (Fig. 5C,D). These results indicated that STZ-mediated accumulation of intracellular Ca^2+^ can influence the progression of STZ-induced AD-like pathology.

### Influence of ROS on STZ-induced AD-like pathology via Ca^2+^/calpain/Cdk5-mediated mitochondrial fragmentation

**Figure 6:**
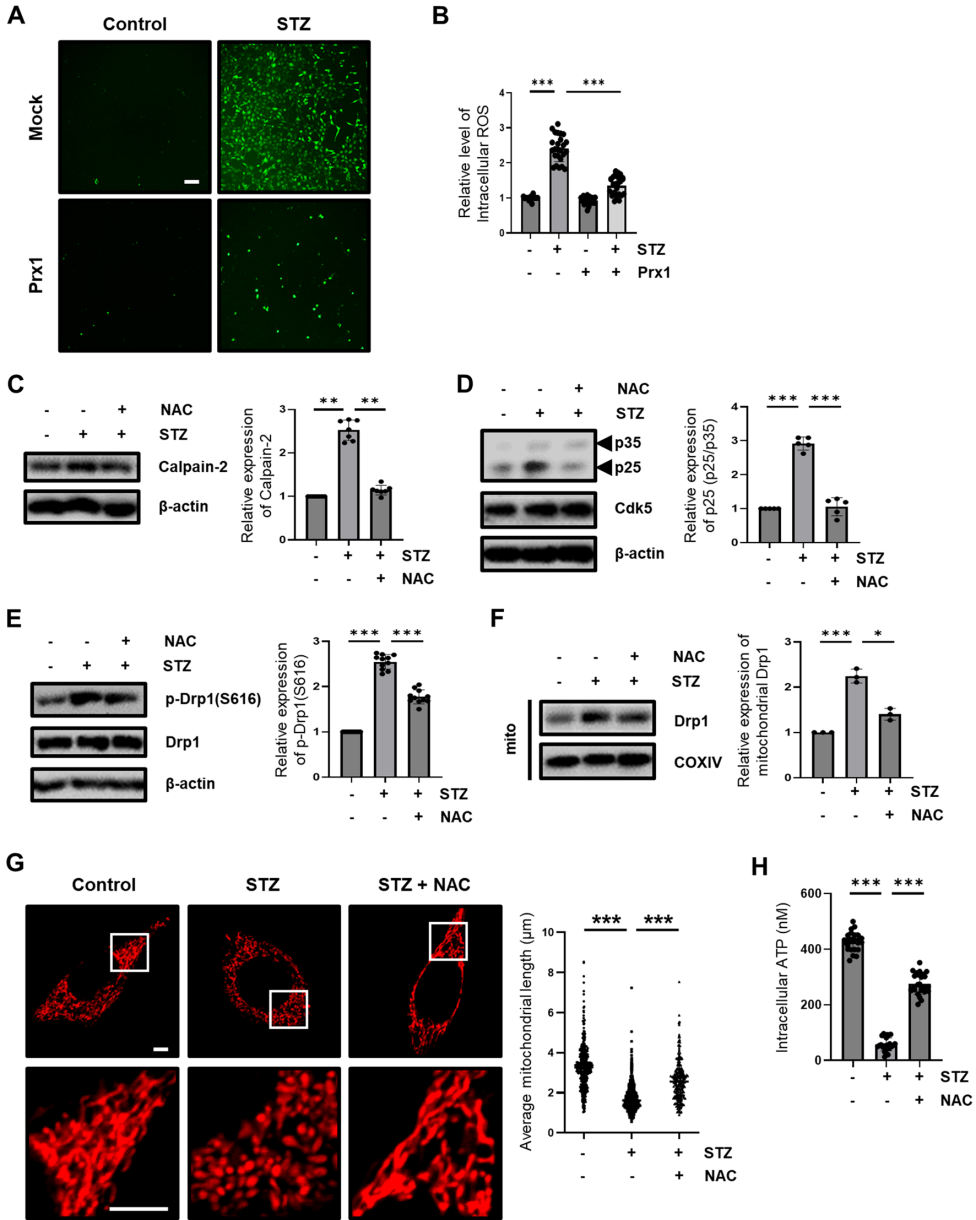
Effect of Prx1 antioxidant capacity on STZ-induced calpain/Cdk5/Drp1 activation. (**A**) Intracellular ROS was observed in STZ (24 h)-treated HT-22 and Prx1-expressed cells with CM-H_2_DCFDA (5 μM) staining using fluorescent microscopy; scale bar, 200 μm. (**B**) Intracellular ROS level was analyzed in STZ (24 h)-treated HT-22 and Prx1-expressed HT-22 cells using flow cytometry with CM-H_2_DCFDA staining. (**C**) Calpain-2 protein expression level in STZ (1.5h)-treated HT-22 cells pretreated with or without NAC (5 mM) were confirmed with western blotting analysis. (**D**) p25/35 and Cdk5 proteins expression level in STZ (6 h)-treated HT-22 cells pretreated with or without NAC were confirmed with western blotting analysis. (**E**) p-Drp1 (S616), (**F**) Mitochondrial Drp1 levels in STZ (12 h)-treated HT-22 cells pretreated with or without NAC were confirmed with western blotting. COXIV was used as the loading control for the mitochondria. (**G**) Change in mitochondrial morphology was observed using confocal microscopy in STZ (12 h)-treated DsRed2-mito expressed HT-22 cells pretreated with or without NAC. The bottom panels showed the magnified images of regions indicated by white squares in the top panels; scale bar, 5 μm. The graph showed distribution of all mitochondrial particles and average mitochondrial length. (**H**) Intracellular ATP levels were measured in STZ (12 h)-treated HT-22 cells pretreated with or without NAC. Blots were cropped to highlight the region of interest; full blot images in this article are provided in supplementary information. The data are presented as mean values ± SD (n ≥ 3). * denotes *p* < 0.05, ** denotes *p* < 0.01, and *** denotes *p* < 0.001.

**Figure 7:**
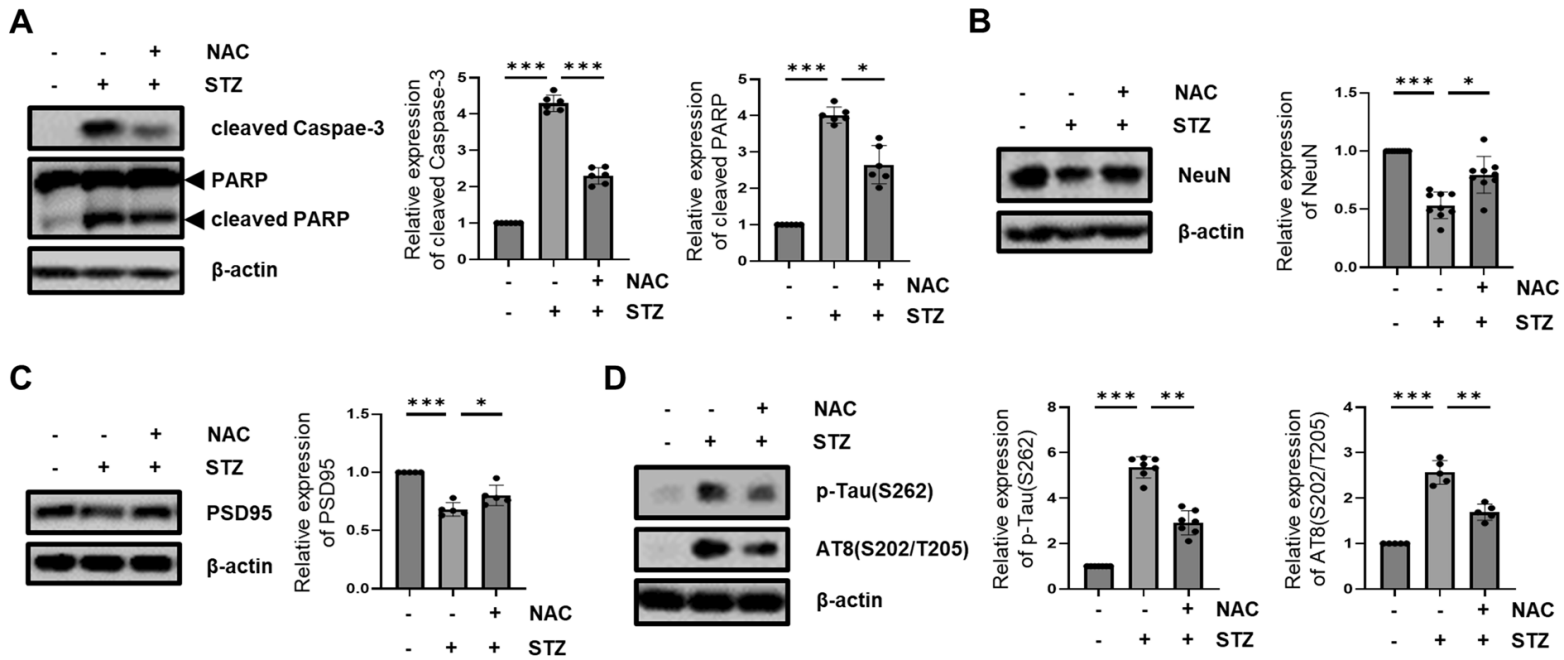
Effect Prx1 antioxidant capacity on STZ-induced AD-like pathology. (**A**) Cleaved Caspase-3 and cleaved PARP, (**B**) NeuN, (**C**) PSD95, (**D**) p-Tau(S262) and AT8(S202/T205) protein expression level were confirmed by western blotting analysis in STZ-treated HT-22 cells pretreated with or without NAC. Blots were cropped to highlight the region of interest; full blot images in this article are provided in supplementary information. The data are presented as mean values ± SD (n ≥ 3). * denotes *p* < 0.05, and *** denotes *p* < 0.001.

Previous reports suggested that oxidative stress relates to the accumulation of intracellular Ca^2+^-mediated calpain activation, and Prx blocks the increase in intracellular Ca^2+^ accumulation^55,56^. Therefore, to assess whether Prx1 affected Ca^2+^-mediated calpain activation using Prx1 antioxidant capacity, we inhibited STZ-induced ROS production using antioxidant molecule, *N*-acetyl-cysteine (NAC). We first determined the effect of Prx1 on STZ-induced intracellular ROS level in STZ-induced HT-22 cells and Prx1-expressed HT-22 cells with CM-H_2_DCFDA. Our results indicated that increased levels of intracellular ROS induced by STZ treatment at 12 h were suppressed by Prx1 expression (Fig. 6A,B). We investigated the impact of STZ-induced ROS inhibition on calpain activation and p35 cleavage. Up-regulation of calpain-2 and p25 protein levels was restored by NAC treatment (Figure C and D). Increase of mitochondrial fragmentation and dysfunction, a downstream pathway of Ca^2+^/calpain/Cdk5 was rescued by ROS scavenge through inhibition of Drp1(S616) phosphorylation and mitochondrial location (Fig. 6E,F,G,H). We then assessed the effect of STZ-induced ROS inhibition on the STZ-induced progression of AD-like pathology. Our results showed that STZ-induced increases in neuronal apoptosis, neuronal loss, synaptic loss, and Tau phosphorylation were prevented by NAC treatment (Fig. 7). These results suggested that STZ-induced progression of AD-like pathology in HT-22 cells was involved in increased ROS levels through governing Ca^2+^/Calpain/Cdk5-mediated mitochondrial fragmentation.

## Discussion

In our previous research, we developed an STZ-induced effective and clinically relevant AD-like model in non-human primates and rodents through intra-cisternal magna (ICM) route, characterized by cerebral and hippocampal damage, disintegration of the neurovascular unit, Aβ deposition, neuroinflammation, and Cdk5 activation^57–60^. Recently, we suggested that regulation of Cdk5/Drp1-dependent mitochondrial morphology plays a role in the potential inhibitor of abnormal metabolic functions associated with AD-like pathogenesis^53^. Based on these findings, a more detailed molecular mechanistic investigation of the STZ-induced AD-like pathologies is warranted.

Oxidative stress precedes the onset of significant AD pathology in the brains of patients and animal models with AD^61,62^. Antioxidant defense systems in the response to oxidative stress, similar to changes in the expression of Prx subtypes seem to be involved in AD pathology, but this remains controversial^63–66^. Mounting evidence determined that various types of Prxs, such as Prx5 and Prx6 have been associated with regulation of the progress of AD pathologies^33,54,67^. Specifically, Prx1 was mainly expressed in oligodendrocytes and astrocytes and was detected in a few neuronal cells^68,69^. However, Prx1 expression was increased in an Aβ-resistant neuronal cell line response to oxidative stress^63^. Therefore, we focused on the role of Prx1 to assess the association with an STZ-mediated antioxidant response in HT-22 hippocampus cell line. We found that Prx1 was upregulated in a time-dependent manner in response to STZ-mediated oxidative stress. However, it was confirmed that although the expression of Prx1 increased by twofold 24 h after STZ treatment, it was insufficient to prevent the progression of AD-like pathology and cell death. Therefore, to find out the role of Prx1 in increasing by STZ treatment, we produced the Prx1 overexpression cell line in HT-22 cells to assume a situation in which Prx1 was expressed at an early timepoint of STZ treatment.

Prx1 overexpression inhibited STZ-mediated neuronal apoptosis, synaptic loss, and tau phosphorylation by preventing cellular ROS accumulation. Furthermore, STZ-mediated mitochondrial fragmentation was suppressed by Prx1 overexpression through the prevention of Cdk5-dependent Drp1 phosphorylation. Our results suggested that Prx1 suppressed neuronal apoptosis by inhibiting the increase of cleaved caspase-3. Previous research demonstrated that caspase-3 activation was regulated by mitochondrial morphology in conditions of neuronal death^70–72^. Furthermore, amount studies found that Prx1 was a key inhibitor of the caspase-3 activation in neuronal death states^73,74^. It has been suggested that dysregulation of Cdk5 homeostasis has pathological relevance to AD^46,75–77^. Oxidative stress is considered to be a crucial modulator of Cdk5 activation^78,79^. Prx5 was involved in Cdk5 activation by modulating oxidative stress^80,81^, and Prx1 activation inhibited Aβ-induced impaired axonal transport^82^. However, the precise relationship between Prx1 and Cdk5 activation in STZ-mediated AD-like pathogenesis are still unclear. Our findings reveal that Prx1 is an important suppressor of STZ-induced progression of AD-like pathology via Cdk5 activation and mitochondrial fragmentation.

Cdk5 activation by p25 is triggered by the activation of calpain, which is related to an accumulation of intracellular Ca^2+^^79,83^. Our result also showed that the elimination of STZ-induced accumulated intracellular Ca^2+^ suppressed STZ-mediated calpain-2 expression, Cdk5 activation, mitochondrial fragmentation, and progression of AD-like pathology. Oxidative stress is associated with dysregulation of Ca^2+^ release and signal pathway^84,85^, which in turn triggers calpain-2 activation^86–88^. Calpain-2 activation is known to be associated with destabilization of lysosomal membranes and the release of cathepsins in neuronal cytoplasm. Cathepsins releasing by activation of calpain-2-induced lysosomal release dysfunction has developed the calpain-cathepsin hypothesis, and it is associated with mitochondrial dysfunction and neuronal death in AD^89–91^. Oxidative stress is also known to induce cathepsin-mediated cell damage, and inhibition of oxidative stress showed neuroprotective effects^92^. Prx1 modulates Aβ-induced increased intracellular Ca^2+^ level by inhibiting ROS accumulation^82^. Our results suggested that STZ-induced up-regulated Prx1 caused a decrease in Ca^2+^ and calpain-2 levels. Apart from the antioxidant function of Prx1 that reduces peroxides via highly reactive catalytic cysteine oxidation to sulfenic acid^93^, Prx1 displays chaperone function by controlling the protein-binding partners^94^. Therefore, we proved that Prx1 decreased the STZ-induced Ca^2+^-mediated calpain-2 expression depending on Prx1 antioxidant capacity. However, it needs to study the direct role of Drp1 phosphorylation in the process of STZ-induced AD-like pathology. Our results demonstrated that the antioxidant capacity of Prx1 is a key factor in the regulation of Ca^2+^ level and Ca^2+^-dependent calpain-2 expression, Cdk5-related mitochondria fragmentation.

The increased expression level of Prx1 observed in various neurodegenerative conditions^95,96^ and increased expression of Prx1 contribute to resistance to oxidative stress^63^. Prx1 is known to play a protective role against ROS-mediated brain injury, such as endotoxin‑induced injury^97^, Huntington’s disease^98^, and acute ischemic stroke^99^. Furthermore, microglial Prx1 participated in the protective function against endotoxin-induced pro-inflammatory response by regulating oxidative stress^100^, and Prx1 overexpression reduced neuronal inflammation and apoptosis by affecting microglial and astrocyte mRNA stability^95^. Therefore, these findings along with our results suggested that Prx1 plays a protective role in neurodegenerative environments by affecting neuron, astrocyte, and microglia.

Apart from these results, our findings have the limitation that it does not reflect complex environment present in the brains of AD rodents or patients because these findings were investigated from an immortalized hippocampal cell line, HT-22 cells. Furthermore, our study did not address the cause of Prx1 increase by STZ treatment. It is known that many transcription factors are involved in the induction of Prx1, and it is very important to study the mechanism of Prx1 expression by STZ treatment in the hippocampus cell line^101–103^. The stability of Prx1 is also one of the factors to consider. Research is needed to determine whether the reason why the expression of Prx1 increases after 12 h due to STZ treatment is due to the expression of Prx1 or changes in factors that regulate the stability of Prx1, such as ubiquitination^104–106^. Although several advantages of HT-22 cells^107–109^, such as AD pathology being well reflected or useful for molecular mechanism study, more research should be conducted in primary hippocampus cells or AD animal models based on the results of this study. Consequently, regulation of Prx1 may be a potential inhibitor of STZ-induced neurodegeneration by preventing the Ca^2+^/capain-2/Cdk5 signal pathway and may be considered as a possible strategy for developing therapies to treat the pathogenesis of AD.

### Supplementary Information

Supplementary Figures.

## Data Availability

All the data generated and/or analyzed during performing this current study are included in this article [and also in its supplementary dataset files]. However, there is no restriction on the availability of materials and data from the corresponding author on reasonable request.
