# Supplementary material for: Peroxiredoxin 1 inhibits streptozotocin-induced Alzheimer’s disease-like pathology in hippocampal neuronal cells via the blocking of Ca2+/Calpain/Cdk5-mediated mitochondrial fragmentation
Source: Sci Rep. 2024 Jul 8;14:15642. doi: 10.1038/s41598-024-66256-x (PMC11231305; doi:10.1038/s41598-024-66256-x)

Supplementary information

Original blots:

Figure 1 (The red boxes represent the blots used in the manuscript)


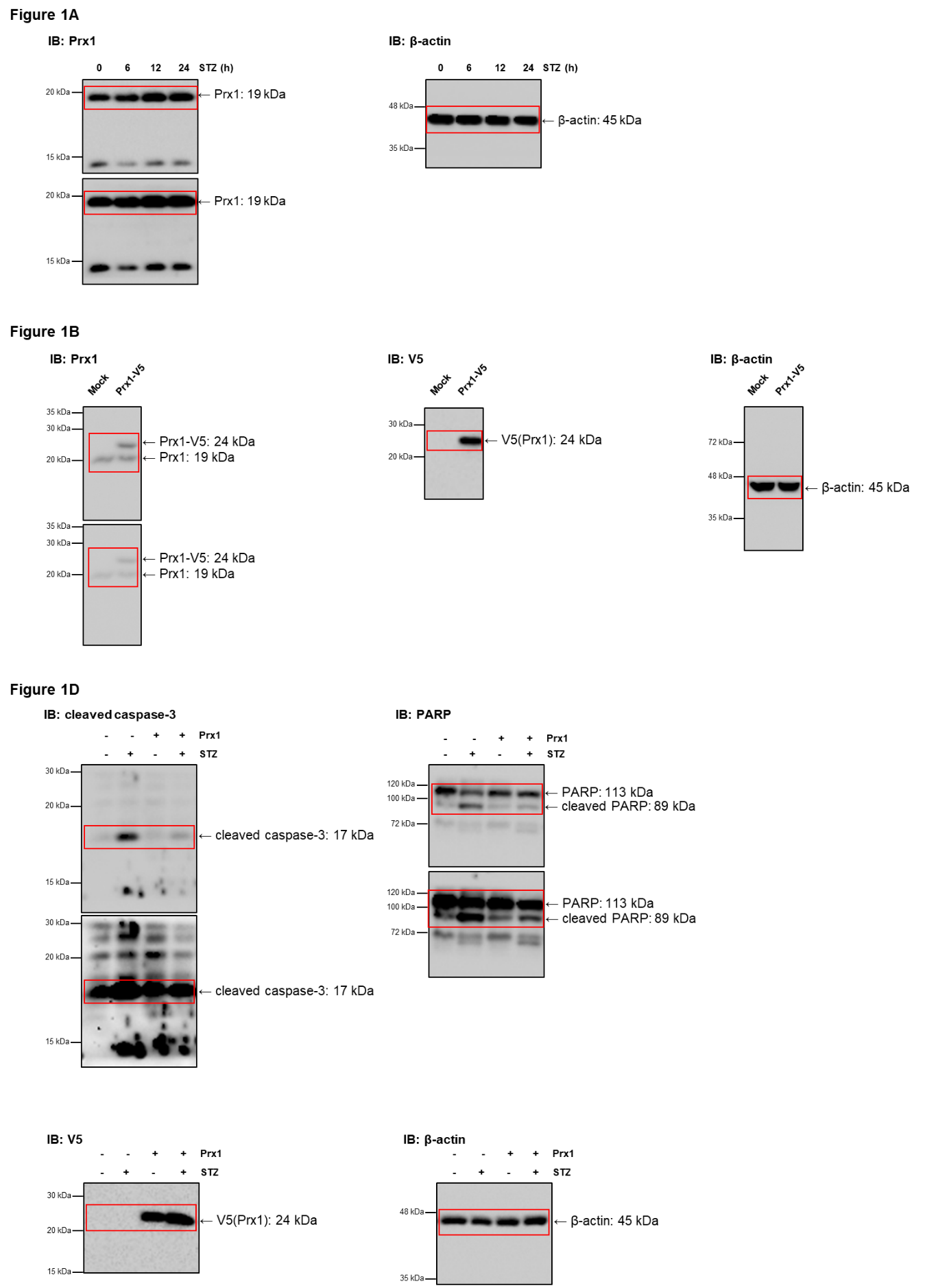


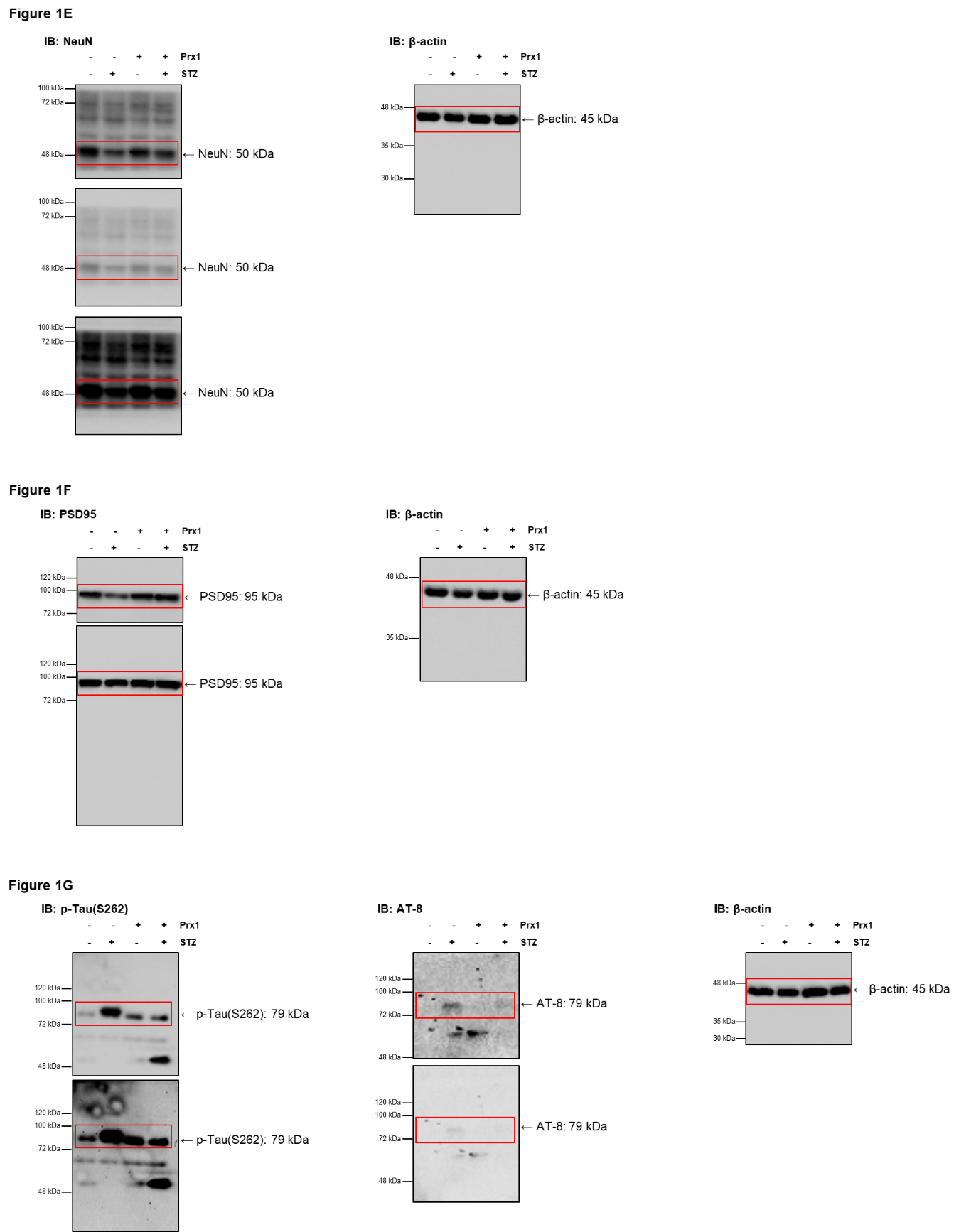


Figure 2 (The red boxes represent the blots used in the manuscript)


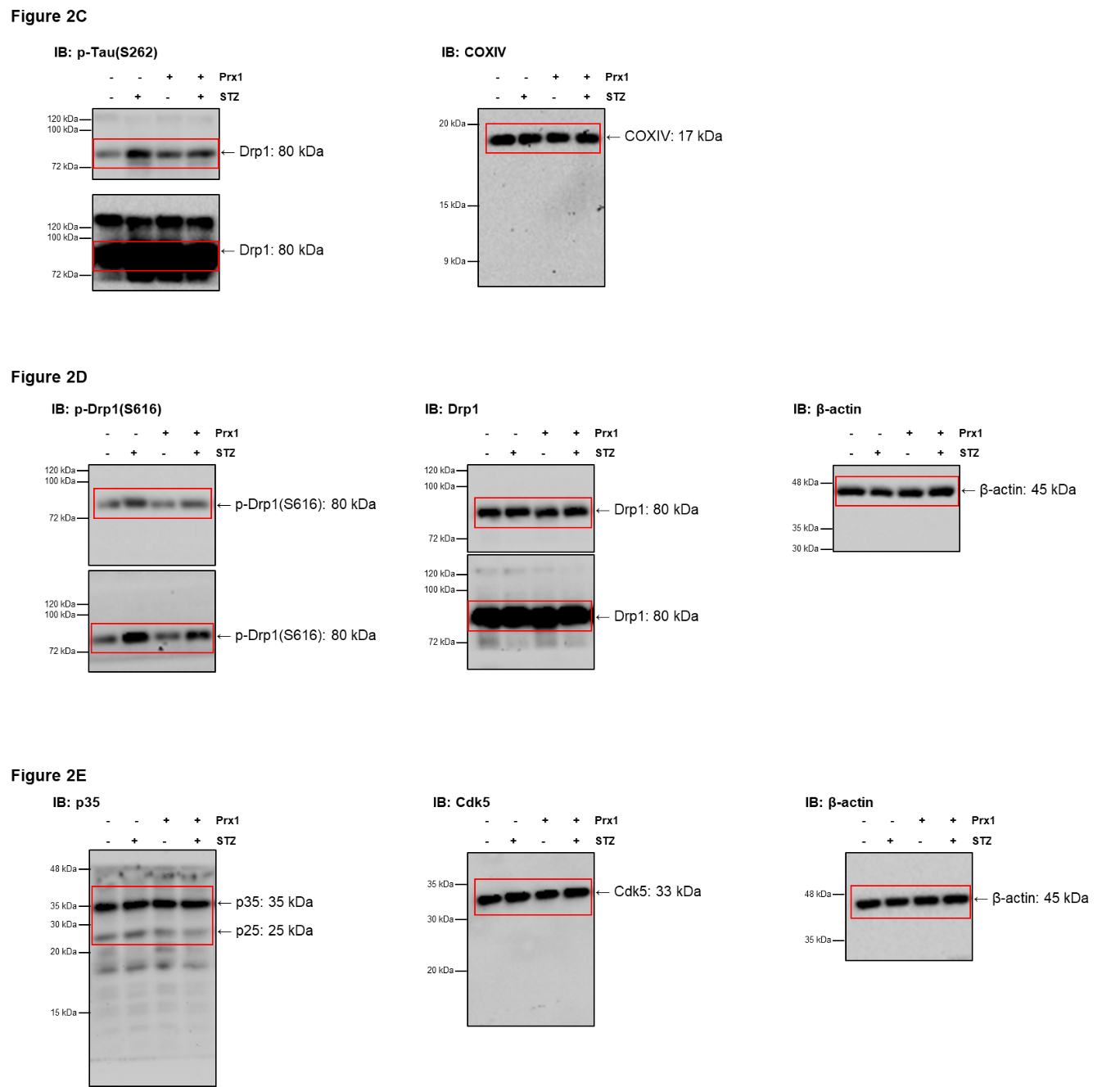


Figure 3 (The red boxes represent the blots used in the manuscript)


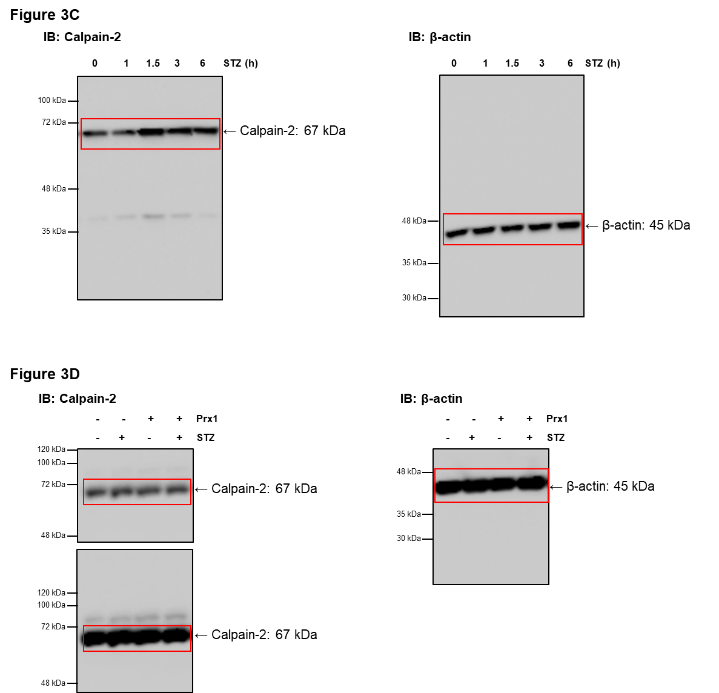


Figure 4 (The red boxes represent the blots used in the manuscript)


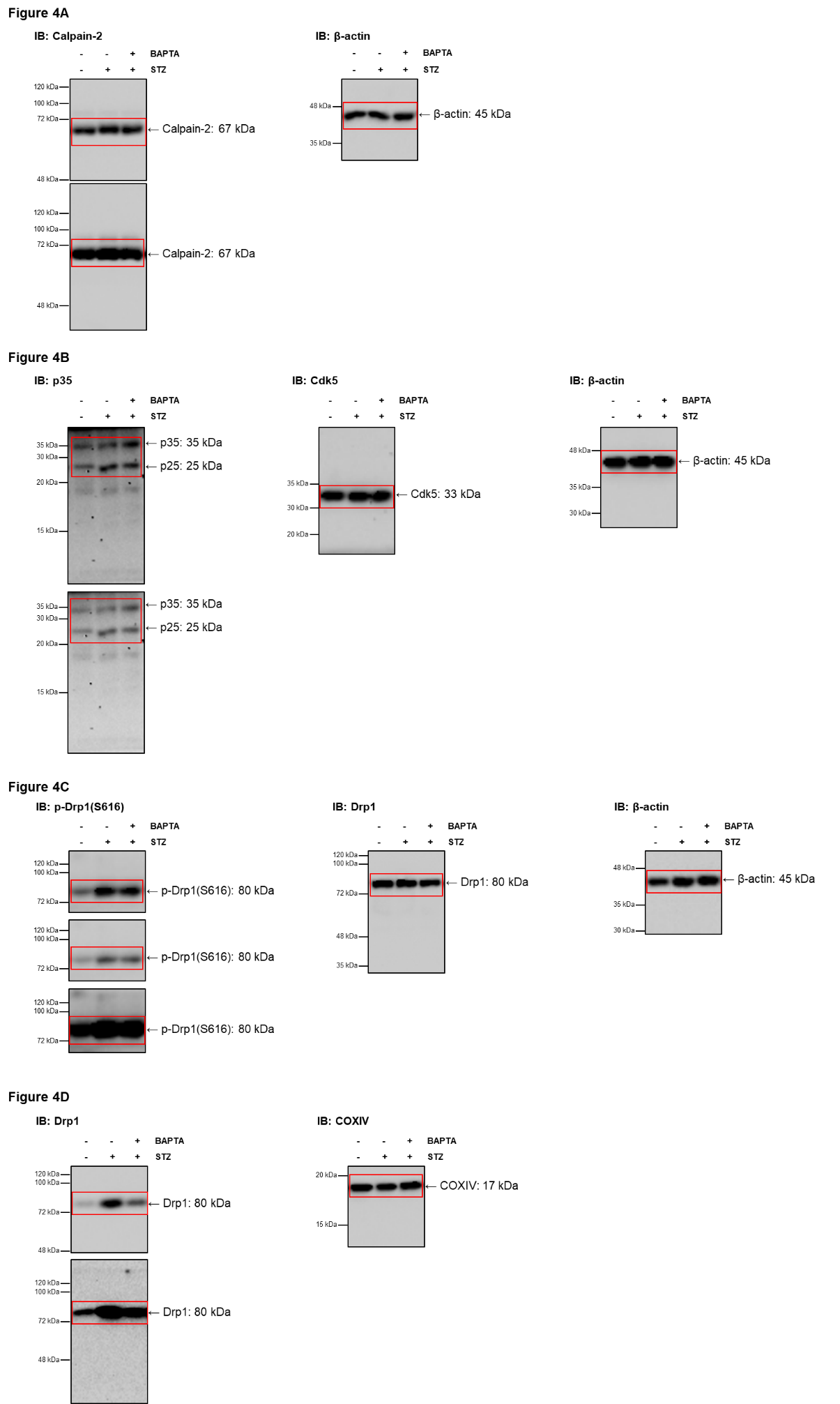


Figure 5 (The red boxes represent the blots used in the manuscript)


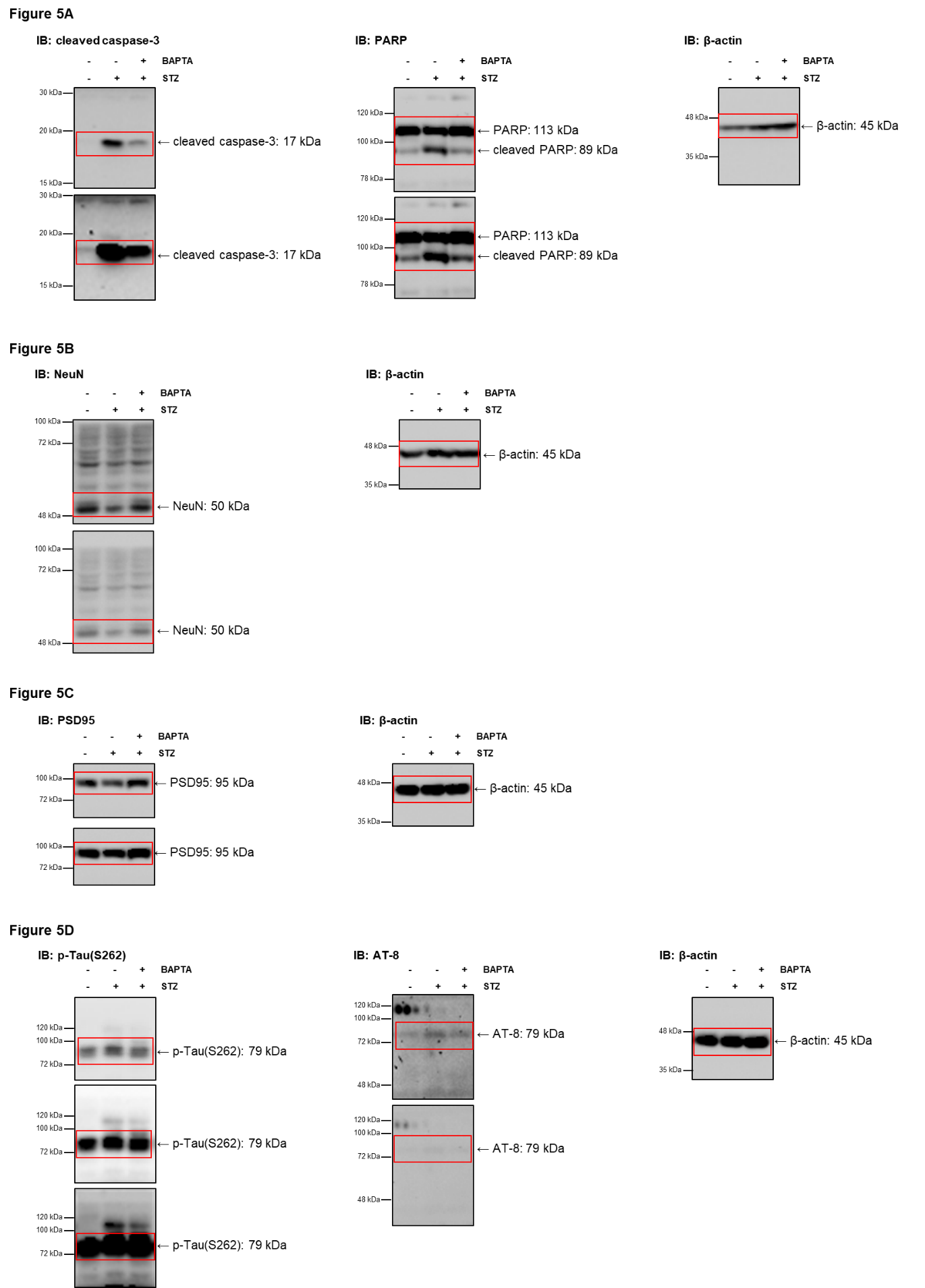


Figure 6 (The red boxes represent the blots used in the manuscript)


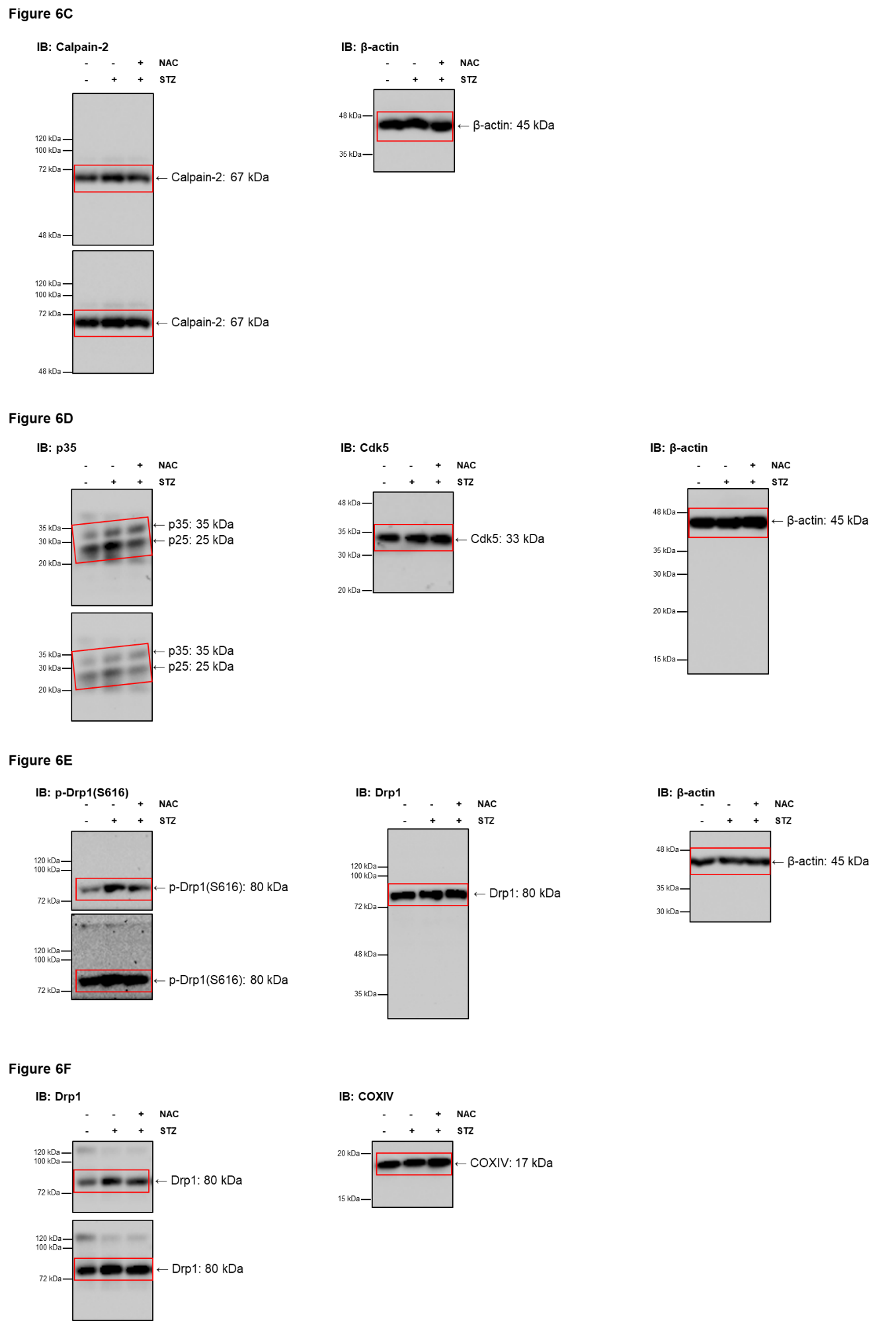


Figure 7 (The red boxes represent the blots used in the manuscript)


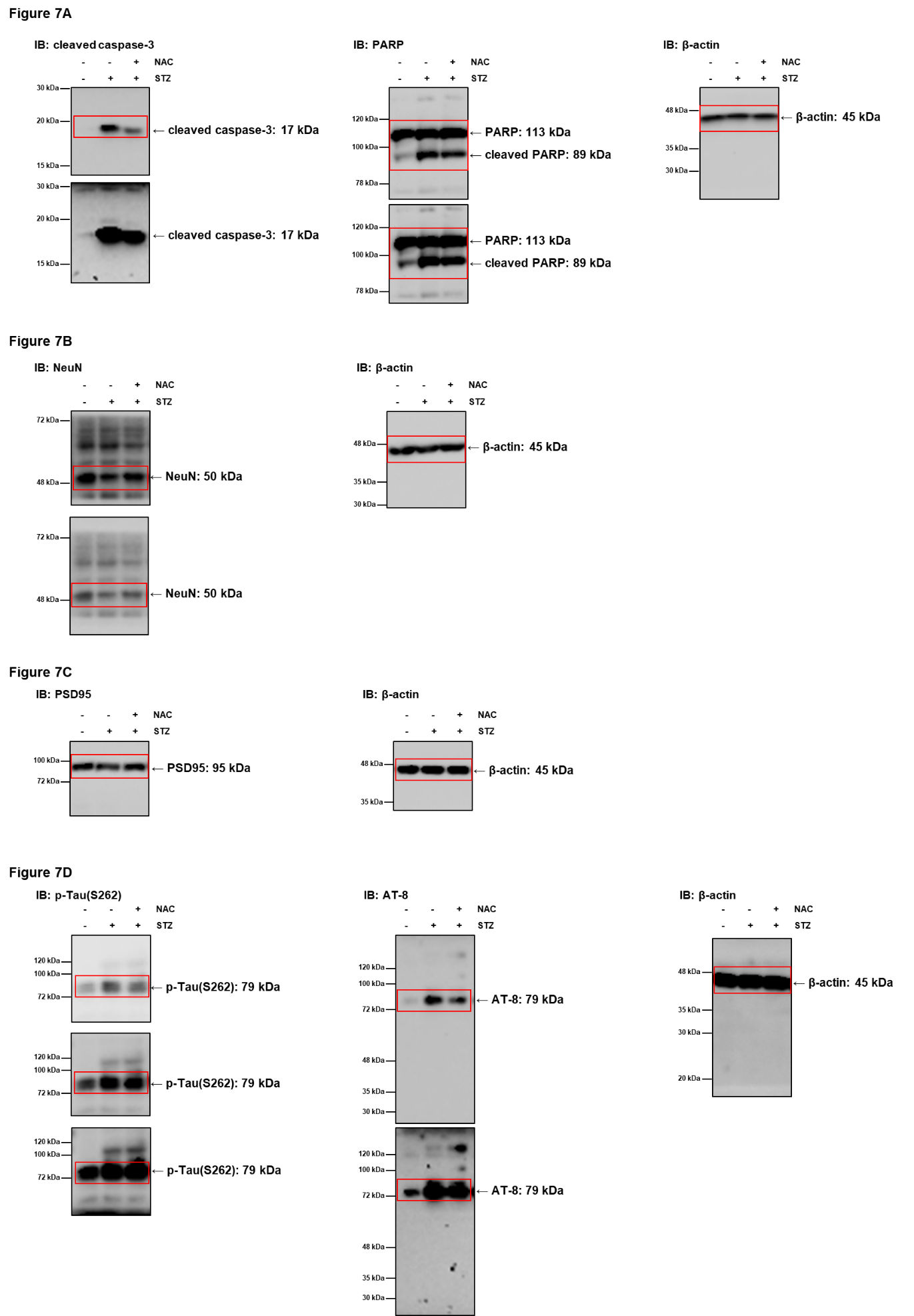

Supplement: Supplementary file 1 — Supplementary Figures. [file 41598_2024_66256_MOESM1_ESM.docx]
